# Yerba Mate (*Ilex paraguariensis A. St.‐Hil.*) Reduces Oxidative Stress and Bone Resorption in Apical Periodontitis

**DOI:** 10.1111/iej.70031

**Published:** 2025-09-16

**Authors:** Carolina Sayuri Wajima, Carolina de Barros Morais Cardoso, Caroline Anselmi, Renan Dal‐Fabbro, Murilo Catelani Ferraz, Cristiane Cantiga da Silva, Paulo César Ciarlini, Edilson Ervolino, Marco Cícero Bottino, Luciano Tavares Angelo Cintra

**Affiliations:** ^1^ Department of Restorative Dentistry São Paulo State University (UNESP), School of Dentistry Araçatuba Brazil; ^2^ Department of Morphology and Pediatric Dentistry São Paulo State University (UNESP), School of Dentistry Araraquara Brazil; ^3^ Department of Cariology, Restorative Sciences, and Endodontics, School of Dentistry University of Michigan Ann Arbor Michigan USA; ^4^ Department of Animal Clinic, Surgery and Reproduction São Paulo State University (UNESP), School of Veterinary Araçatuba Brazil; ^5^ Department of Basic Science São Paulo State University (UNESP), School of Dentistry Araçatuba Brazil; ^6^ Department of Biomedical Engineering, College of Engineering University of Michigan Ann Arbor Michigan USA

**Keywords:** bone loss, bone remodelling/regeneration, cytokine(s), endodontics, herbal medicine, host modulation therapy

## Abstract

**Aim:**

Apical periodontitis (AP) is a highly prevalent chronic inflammatory disease that can exert systemic effects by releasing biochemical mediators that initiate and regulate the immune response. Yerba Mate ( *Ilex paraguariensis* , YM), a popular plant broadly consumed in South America, is rich in biologically active compounds known for their therapeutic potential. This study assessed Y's in vitro cytocompatibility, anti‐osteoclastogenic and immunomodulatory effects on oral stem cells and macrophages, as well as its in vivo potential to reduce AP severity and systemic side effects.

**Methodology:**

In vitro, instant powdered YM was dissolved in distilled water, filtered and diluted in culture media to final concentrations ranging from 1 to 200 μg/mL. Cell viability and pro‐inflammatory cytokine release (IL‐1α, IL‐6, TNF‐α) were assessed in human exfoliated deciduous teeth stem cells. The NF‐κB pathway and anti‐osteoclastogenic activity were evaluated using a luciferase reporter assay and TRAP staining in RAW 264.7. Forty male Wistar rats were divided into control (C), YM‐treated(YM), AP‐induced (AP) and AP with YM treatment (AP + YM). YM treatment (20 Mg/Kg/Day) was administered via gavage for 58 days. AP was induced after 28 days of YM intake, and the animals were euthanised 30 days later. In serum, systemic redox state was assessed via total antioxidant capacity (TAC) and thiobarbituric acid reactive substances (TBARS). Histological and immunohistochemical analyses evaluated inflammation, cytokine expression (TNF‐α, IL‐6, IL‐10 and IL‐17) and bone resorption markers. Micro‐CT quantified alveolar bone loss. Data were analysed at *p* < 0.05.

**Results:**

YM treatment demonstrated significant anti‐inflammatory, antioxidant and bone‐protective effects. In vitro, YM‐supported cell viability, reduced TNF‐α and IL‐1α, inhibited NF‐κB activation and suppressed osteoclastogenesis. In vivo, YM treatment restored systemic antioxidant capacity and reduced lipid peroxidation, mitigating AP‐induced oxidative stress. Furthermore, YM intake attenuated the local inflammatory response and reduced the bone resorptive activity associated with AP.

**Conclusion:**

In vitro, YM suppressed pro‐inflammatory cytokines and NF‐κB, inhibited osteoclastogenesis and was cytocompatible. In vivo, it reversed AP‐induced redox state and reduced inflammation and bone resorption, suggesting therapeutic promise.

## 1 Introduction

Apical periodontitis (AP) is marked by inflammation and destruction of the tissues surrounding the tooth's apex, primarily driven by polymicrobial infections originating from the tooth itself and exacerbated by the host's immune response, which contributes to extracellular matrix breakdown and bone loss (Tiburcio‐Machado et al. 2021). Affecting nearly half of the global population, AP is a significant public health concern due to the impact of oral infections on broader health challenges, as this localised condition can also have systemic effects and potentially contribute to the development of other diseases (Segura‐Egea et al. 2015; Cintra et al. 2021; Tiburcio‐Machado et al. 2021).

With the recent growth of research in endodontic medicine, which explores the interrelationship between systemic conditions and periapical tissue pathologies, it is well established that AP acts as a chronic condition that continuously releases inflammatory cells and mediators into the bloodstream that can exacerbate systemic diseases in vulnerable areas (Segura‐Egea et al. 2015; Cintra et al. 2021). On the other hand, researchers have revealed that certain compounds in dietary supplements can mitigate periapical inflammation and reduce these systemic effects of AP's immunoinflammatory response, highlighting the potential for complementary interventions to be used with conventional root canal therapies targeting local and systemic impacts of AP (Azuma et al. 2018; Cosme‐Silva et al. 2019; Justo et al. 2022).

Yerba mate ( *Ilex paraguariensis* , A. St. Hil., 1822) (YM) is an evergreen tree native to South America, renowned for its medicinal properties and complex chemical composition (Bracesco et al. 2011). Rich in phenolics, saponins, methylxanthine alkaloids, carotenoids, minerals and vitamins, YM has been employed in developing new health products (Heck and de Mejia 2007; Blum‐Silva et al. 2016). Its consumption offers several beneficial physiological effects, including reduced risks of cardiovascular diseases and diabetes (Cardozo Junior and Morand 2016). Additionally, YM exhibits immunomodulatory, anti‐inflammatory (Luz et al. 2016), antimicrobial (Kungel et al. 2018), antioxidant (Wu et al. 2017) and chemopreventive properties (Cittadini et al. 2019). Due to its high content of phenolic compounds that may protect against chronic diseases, such as AP, this plant has significant pharmacological and clinical interest, evidenced by the increasing number of patent applications in recent years for products containing *Ilex paraguariensis* (Grando et al. 2017).

In dentistry, YM has demonstrated antimicrobial activity against some of the main odontogenic microorganisms, such as *Staphylococcus aureus* (Fayad et al. 2020), *Streptococcus mutans* and *Enterococcus faecalis* (Noureddine et al. 2018). Moreover, YM promotes bone neoformation and reduces inflammatory infiltration during alveolar healing (Brasilino et al. 2018). Although some evidence supports its use in dentistry, no studies have assessed YM's potential for endodontics. Here, we evaluate the YM on in vitro cytocompatibility, anti‐osteoclastogenic and immunomodulatory effects on oral‐derived stem cells and macrophages, as well as its in vivo potential to modulate the severity of AP and counteract the systemic side effects that this condition evokes.

## 2 Materials and Methods

**FIGURE 1:**
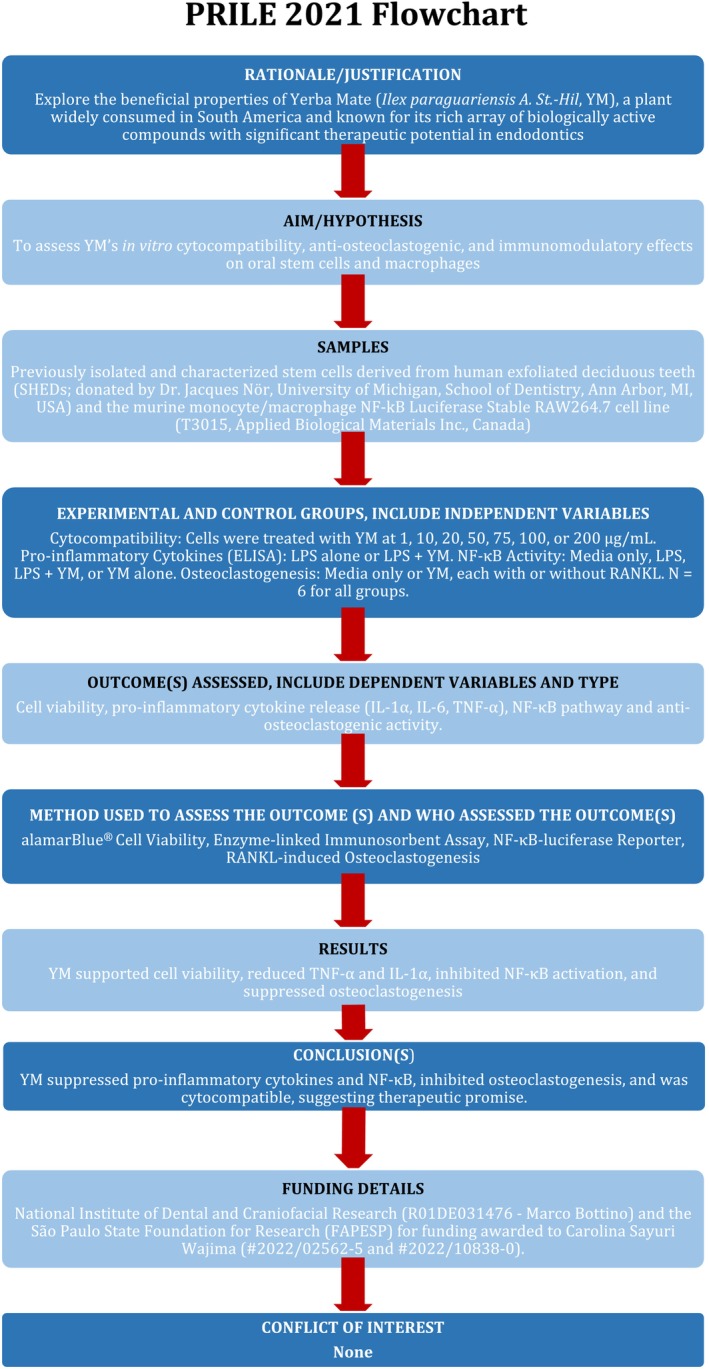
PRILE flowchart.

**FIGURE 2:**
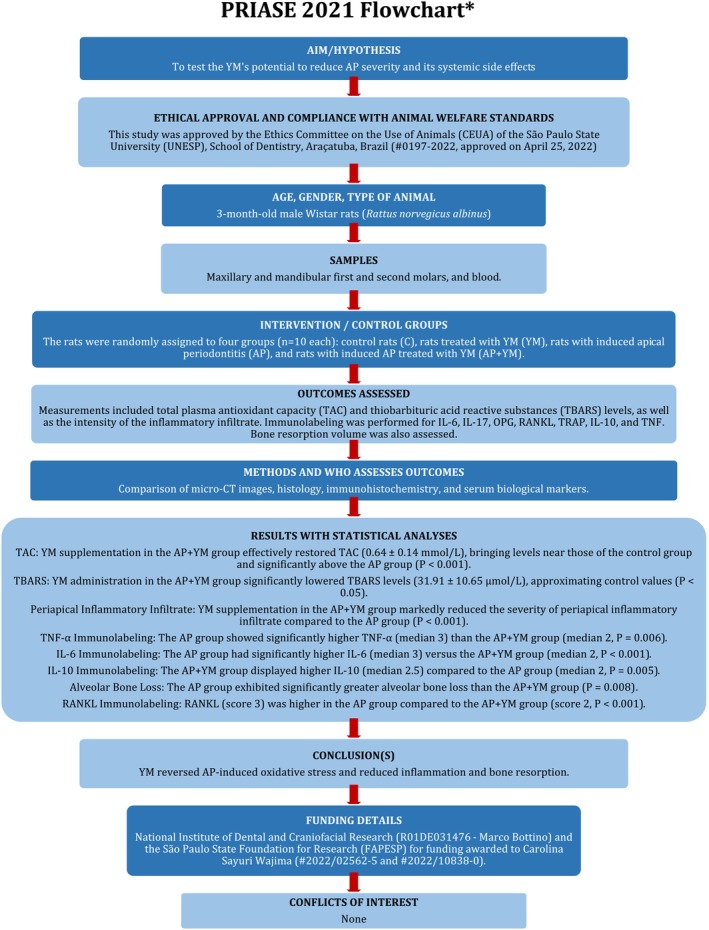
PRIASE flowchart.

The manuscript of this laboratory study has been written according to the Preferred Reporting Items for Laboratory Studies in Endodontology (PRILE) 2021 Guidelines (Figure 1). The manuscript of this animal study has been written according to the Preferred Reporting Items for Animal Studies in Endodontology (PRIASE) 2021 Guidelines (Figure 2).

### 2.1 Yerba Mate's Preparation for Cell Culture

Instant powdered YM (Leão Jr., Curitiba, PR, Brazil, batch #140122) was used in this study. To prepare a stock solution at a concentration of 20 mg/mL, 200 mg of the powder was dissolved in 10 mL of distilled water heated to 80°C. The solution was then filtered (0.22 μm pore size; Durapore, GVWP04700, Sigma‐Aldrich, St. Louis, MO, USA). This stock solution was subsequently diluted with α‐MEM (+L‐Glutamine, +Ribonucleosides, +Deoxyribonucleosides, Gibco, Carlsbad, CA, USA) medium without foetal bovine serum (FBS, Gibco) to achieve final concentrations of 1, 10, 20, 50, 75, 100 and 200 μg/mL. All solutions were prepared from the same batch and were freshly made and used immediately after each change of the culture medium (Balera Brito et al. 2019).

Two cell lines were used in this study. Stem cells derived from human exfoliated deciduous teeth (SHEDs; Lonza, Walkersville, MD, USA) were employed for the cell viability and pro‐cytokine release assays. SHEDs are immature and multipotent highly proliferative mesenchymal stem cells with fibroblastic morphology (Miura et al. 2003). Passages 4–8 were used, and these cells were cultured at 37°C in a 5% CO_2_ incubator using α‐MEM supplemented with 15% FBS and 1% penicillin–streptomycin (All from Gibco). The murine monocyte/macrophage NF‐kB Luciferase Stable RAW264.7 cell line (T3015; Applied Biological Materials Inc., Canada) was utilised for NF‐κB luciferase activity assays and RANKL‐induced osteoclastogenesis studies. Passages 3–8 were used, and these cells were cultured under the same conditions in D‐MEM supplemented with 10% FBS and 1% penicillin–streptomycin (all from Gibco).

### 2.2 alamarBlue Cell Viability

For the cell viability assay (*n* = 4), after the SHEDs reached 80%–90% confluence, they were seeded in 96‐well plates at a density of 1 × 10^4^ cells/well. Twenty‐four hours after seeding, when the confluence was between 80% and 90%, the cells were treated with increasing concentrations of YM (1, 10, 20, 50, 75, 100 and 200 μg/mL). α‐MEM without FBS was used as a control (C). The medium was refreshed every 2 days. Cell viability was evaluated using the alamarBlue assay (Invitrogen, Carlsbad, CA, USA) at 1, 3 and 5 days. Cells were incubated in α‐MEM containing alamarBlue reagent (10:1) at 37°C and 5% CO_2_. After 3 h, the fluorescence signal of 100 μL of the supernatant was measured at 560 nm excitation and 590 nm emission using a SpectraMax iD3 plate reader (Molecular Devices LLC, San Jose, CA, USA). Fluorescence values were calculated as a percentage of the control group at each time point, with the control set as 100% (Anselmi et al. 2022).

### 2.3 Enzyme‐Linked Immunosorbent (ELISA) Assay

For ELISA assay (*n* = 4), after the SHEDs reached 80%–90% confluence, they were seeded in 96‐well plates at a density of 3 × 10^4^ cells/well in complete α‐MEM. After 24 h, the cells were stimulated with *Escherichia coli* lipopolysaccharide (LPS, serotype O111:B4, 10 μg/mL, 100 μL/well; Sigma‐Aldrich, St. Louis, MO) for 7 days, refreshed every 2 days. After the LPS stimulus, cells were treated or not with 50 μg/mL YM, the highest non‐cytotoxic concentration determined in the prior assay. After 24 h, culture supernatants were collected, and concentrations of IL‐1α (BioLegend Kit 433 404), IL‐6 (BioLegend Kit 431 304) and TNF‐α (BioLegend Kit 430 904) were measured using the respective ELISA kits according to the manufacturer's instructions.

### 2.4 NF‐κB‐Luciferase Reporter Assay

The effect of YM extract on the nuclear factor kappa B (NF‐κB) signalling pathway was evaluated using a reporter assay with NF‐κB luciferase‐stable RAW 264.7 macrophage‐like cells (*n* = 6). Cells were seeded at a density of 7.5 × 10^4^ cells per well in 96‐well plates and incubated for 24 h for initial adhesion. Then, cells were stimulated with 0.1 μg/mL of LPS and treated with or without 50 μg/mL YM. Cells untreated with YM or LPS stimulus were used as controls. After 6 h, NF‐κB activation was quantified using a luciferase assay system (Promega, Madison, WI, USA). Luciferase activity was measured with a SpectraMax iD3 plate reader, performing a 2 s delay, followed by a 10 s reading for luminescence (Dal‐Fabbro et al. 2024).

### 2.5 RANKL‐Induced Osteoclastogenesis

To assess the anti‐osteogenic potential of YM extract (*n* = 6), RAW 264.7 cells were seeded in 96‐well plates at a density of 3 × 10^3^ cells/well. The cells were cultured with or without 50 ng/mL RANKL (#462‐TR, R&D Systems, USA), either alone or in the presence of YM extract (50 μg/mL). The medium was refreshed every 2 days. After 5 days, the cells were stained for tartrate‐resistant acid phosphatase (TRAP) using a leukocyte acid phosphatase kit (Sigma‐Aldrich). TRAP‐positive cells with more than three nuclei were identified as osteoclasts and counted. The results were expressed as the number of osteoclasts per well.

### 2.6 Animals

This study was approved by the Ethics Committee on the Use of Animals (CEUA) of São Paulo State University (UNESP), School of Dentistry, Araçatuba, Brazil (#0197–2022; approved on April 25, 2022). The study adhered to the Animal Research: Reporting of In Vivo Experiments (ARRIVE) guidelines and followed the National Institutes of Health Guide for the Care and Use of Laboratory Animals (Percie du Sert et al. 2020). The sample size was determined based on data from previous studies conducted by our research group, considering a 5% alpha error and 95% statistical power to detect a significant median difference of 1 (Cosme‐Silva et al. 2019; Dal‐Fabbro et al. 2019; Percie du Sert et al. 2020).

Forty 3‐month‐old male Wistar rats (*Rattus norvegicus albinus*) weighing 230–260 g were housed in mini‐isolators (Alesco, Monte Mor, São Paulo, Brazil) under controlled conditions, including a temperature of 22°C–24°C and a 12‐h light/dark cycle. They were provided a solid diet and water *ad libitum* throughout the experimental period. The rats were randomly assigned to four groups (*n* = 10 each): control rats (C), rats treated with YM (YM), rats with induced apical periodontitis (AP) and rats with induced AP treated with YM (AP + YM).

### 2.7 Yerba Mate's Treatment

Daily oral treatment was performed using the gavage technique, administering instant powdered tea (Leão Jr.) at a 20 mg/kg dose. The tea was freshly prepared daily by dissolving the powder in 0.5 mL of pure water immediately before administration, a dosage equivalent to consuming 300 mL (1.5 cups) of YM tea daily for humans (Pereira et al. 2017; Brasilino et al. 2018). The YM and AP + YM groups were treated for 58 consecutive days. The treatment was considered prophylactic during the first 28 days before the AP induction, simulating routine consumption. Following AP induction, treatment continued for an additional 30 days until euthanasia, during which it was considered therapeutic. Animals in the control (C) and AP groups received the same volume of pure water and underwent the exact handling to account for stress. The animals were weighed daily using an electronic scale before preparing the YM extract to ensure accurate dose adjustments for each animal. Body weight gain was monitored by calculating the percentage change in weight, comparing measurements taken on days 7, 14, 21, 28, 35, 42, 49 and 58 to the initial weight.

### 2.8 Apical Periodontitis Induction

On the 28th day of the experiment, the animals were anaesthetised intramuscularly with a combination of 2% xylazine (10 Mg/Kg; Xilazin, Syntec Do Brasil LTDA, Cotia, São Paulo, Brazil) and 10% ketamine hydrochloride (80 Mg/Kg; Ketamine Agenor 10%, União Química Farmacêutica Nacional S/A, Embu‐Guaçu, São Paulo, Brazil). The pulps of the maxillary and mandibular first and second molars on the right side were then exposed using a 0.5 mm diameter carbon steel drill (Ln Long Neck‐Maillefer Drill, Dentsply) and left open to the oral cavity for 30 days until euthanasia (Cintra et al. 2016). Post‐surgery, animals received subcutaneous administration of a non‐steroidal anti‐inflammatory drug (5 mg/kg^−1^ Carprofen, Pfizer).

### 2.9 Systemic Biochemical Analysis

To assess total plasma antioxidant capacity (TAC) and thiobarbituric acid reactive substances (TBARS) levels in serum, venous blood samples (5 mL) were collected via cardiac puncture from anaesthetised animals 30 days after the AP induction. The blood was centrifuged at 1800 × ***g*** for 15 min at 4°C to separate the serum. The total antioxidant capacity of plasma was assessed using the Ferric reducing antioxidant power (FRAP) assay with a commercial kit (Exodus Scientific; CCK072‐20). This method detects non‐enzymatic antioxidants through the ability to reduce ferric ions (Fe^3+^) to ferrous ions (Fe^2+^) by antioxidants present in the plasma. The change in absorbance at 593 nm was measured and compared to a standard curve for quantification (Brasilino et al. 2018). Also, serum lipid peroxidation was determined by quantifying malondialdehyde (MDA) using the modified Hunter method with a commercial kit (Êxodo Científica; CCK023‐100). MDA is formed through β‐cleavage of peroxidised polyunsaturated fatty acids during lipoperoxidation, reacts with thiobarbituric acid (TBA) and is quantified based on absorbance levels at 545 nm (Prieto et al. 2017).

### 2.10 Histological and Immunohistochemical Analysis

Following blood collection, rats were euthanised via intraperitoneal overdose of an anaesthetic. The right hemi‐mandibles were retrieved and fixed in a 10% neutral‐buffered formalin solution for 22 h, then washed in running water for 12 h. The samples were then demineralised in 17% EDTA (pH 8; Sigma‐Aldrich). The lower first molars were sectioned longitudinally at a thickness of 4 μm, focusing on the distal root. Histological analysis was performed on sections stained with haematoxylin and eosin using a light microscope (DM 4000 B; Leica Microsystems, Wetzlar, Germany). A single calibrated, blinded and experienced histologist evaluated the samples based on the nature and extent of necrosis and the cellularity patterns in the dental and periapical tissues. The intensity of the inflammatory infiltrate was classified into four scores: score 0 (no concentration of inflammatory cells), score 1 (fewer than 25 cells, mild reaction), score 2 (25–125 cells, moderate reaction) and score 3 (125 or more cells, severe reaction) (Cosme‐Silva et al. 2019; Dal‐Fabbro et al. 2019).

Immunohistochemical reactions were conducted using the indirect immunoperoxidase technique, as previously described (Cosme‐Silva et al. 2019, Dal‐Fabbro et al. 2019). Primary antibodies were diluted 1:100 and included: anti‐Interleukin 6 (IL‐6) (Rabbit, SC1265), anti‐Interleukin 17 (IL‐17) (Rabbit, SC7927), anti‐Tumour Necrosis Factor‐Alpha (TNF‐α) (Goat, SC1350), anti‐Receptor Activator of Nuclear Factor Kappa‐B Ligand (RANK‐L) (Goat, SC7627), anti‐Osteoprotegerin (OPG) (Rabbit, SC11383) and anti‐Tartrate‐Resistant Acid Phosphatase (TRAP) (Goat, SC30832) from Santa Cruz Biotechnology (Santa Cruz, CA, USA), as well as anti‐Interleukin 10 (IL‐10) (Rabbit, orb221323; Biorbyt, San Francisco, CA, USA). Immunolabelling was visualised using a biotinylated universal secondary antibody applied for 2 h, followed by a streptavidin–horseradish peroxidase conjugate for 1 h (Universal Dako‐Labelled Streptavidin‐Biotin Kit; Dako Laboratories, Carpinteria, CA, USA). The chromogen 3,3′‐diaminobenzidine (DAB Chromogen Kit; Dako Laboratories) was used for detection, and sections were counterstained with haematoxylin. Negative controls underwent the same procedure but without primary antibody incubation.

Semi‐quantitative immunolabelling analysis was conducted by the same certified histologist using a light microscope (DM 4000 B) equipped with a colour camera (DFC 500; Leica). Positive immunoreactivity (IR) of IL‐6, IL‐10, IL‐17, OPG, RANK‐L and TNF‐α was identified as brownish staining in the cytoplasm of cells and the extracellular matrix in the periapical region. Immunoreactivity was scored according to the following criteria: Score 0 indicated no immunoreactive cells; Score 1 (low IR) corresponded to a few immunoreactive cells and weak labelling of the extracellular matrix, representing approximately 25% of the immunoreactive cells; Score 2 (moderate IR) represented a moderate number of immunoreactive cells and moderate labelling of the extracellular matrix, corresponding to approximately 50% of the immunoreactive cells; and Score 3 (high IR) indicated a large number of immunoreactive cells with intense extracellular matrix labelling, approximately 75% of the immunoreactive cells (Cintra et al. 2016). For TRAP analysis, the perimeter of the bone resorption area associated with AP was outlined, multinucleated TRAP‐positive cells were counted, and their ratio to the resorption perimeter was calculated (Dal‐Fabbro et al. 2019).

### 2.11 Microtomographic Analysis

The right maxillae were retrieved, fixed as previously described and then immersed in 70% alcohol at room temperature. They were scanned using a micro‐CT system (Bruker SkyScan 1272, Aartselaar, Belgium) with the following parameters: 70 kV, 167 μA, 0.5° rotation step, 2100 ms exposure time and 3‐frame averaging. The images were reconstructed using NRecon software (Bruker), composed of a total of 50 slices. The alveolar bone volume loss was measured using CTAn v.1.5.0 software (Bruker), which interpolated a geometric figure to allow for the 3D calculation of volumetric indices in the distal root of the maxillary. The results were expressed in cubic millimetres. Vertically, the volume spanned from the apical foramen to the mid‐root level, corresponding to the middle third. In the axial plane, it was bounded distally by the outer alveolar cortical plate and mesially by the interdental septum (Dal‐Fabbro et al. 2021).

### 2.12 Statistical Analysis

The data were analysed using GraphPad Prism 10 (Boston, MA, USA). The normal distribution of the quantitative continuous variables was checked using the Student's t test and the Shapiro–Wilk test. Data that passed the normality test were analysed using ANOVA and Tukey's multiple comparisons test. Non‐normally distributed data were analysed using the Kruskal–Wallis test, followed by Dunn's method for pairwise comparisons. A significance level of 5% (*p* < 0.05) was applied to all tests.

## 3 Results

### 3.1 Yerba Mate Extract Promotes Cell Viability, Modulates Inflammatory Cytokines and Inhibits Osteoclastogenesis In Vitro

**FIGURE 3:**
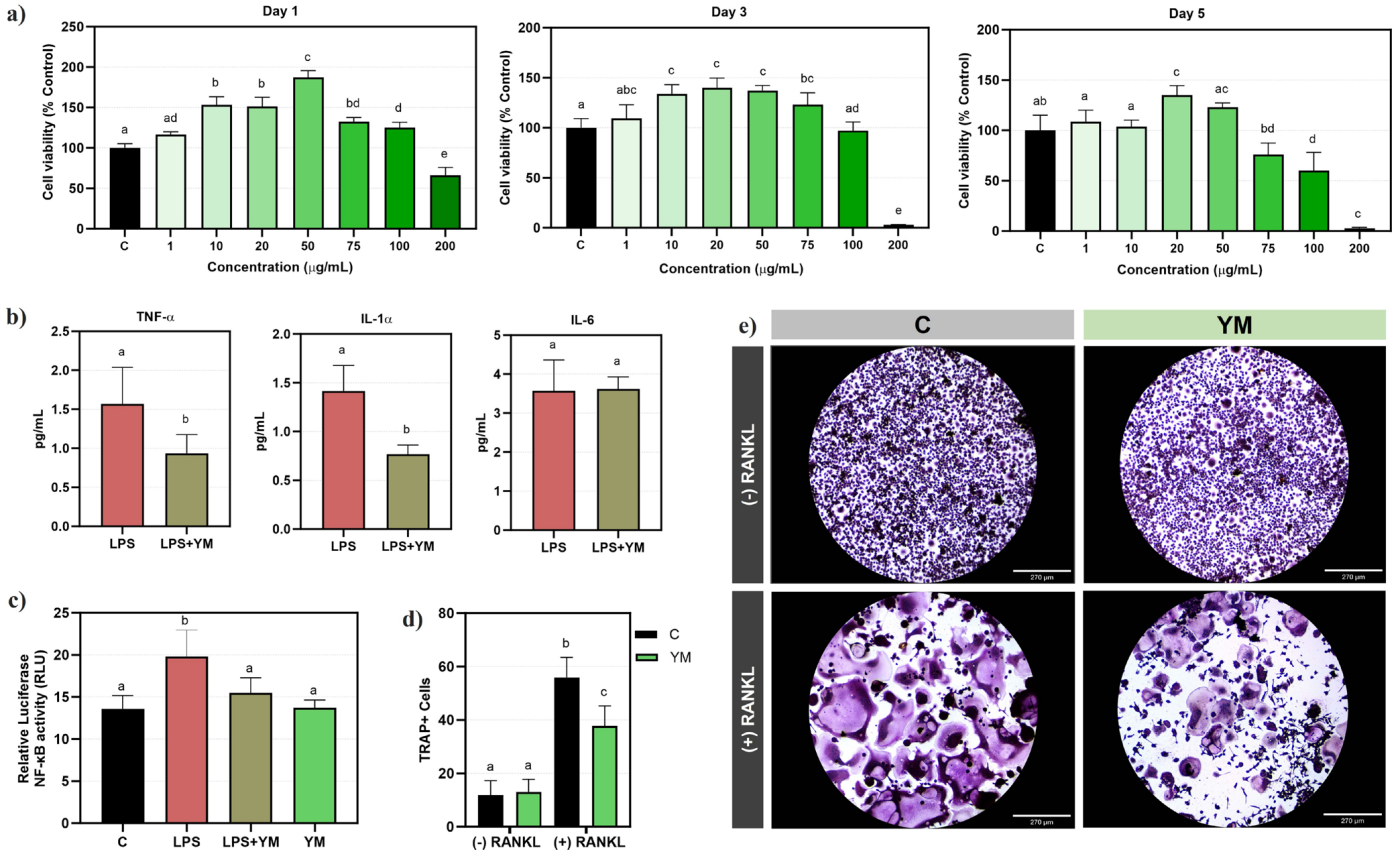
YM extract promotes cell viability, exhibits immunomodulatory properties and suppresses osteoclastogenesis. (a) Cell viability analysis in SHED cells identified 50 μg/mL as the highest non‐cytotoxic dose of YM (*n* = 4). (b) YM significantly reduced the levels of pro‐inflammatory cytokines IL‐1α and TNF‐α in SHED cells (*n* = 4). (c) YM inhibited NF‐κB activation in LPS‐stimulated RAW 264.7 cells, as demonstrated by the NF‐κB luciferase reporter assay (*n* = 6). (d, e) YM suppressed RANKL‐induced osteoclastogenesis in RAW 264.7 cells (*n* = 6). Data are presented as mean ± standard deviation. Statistical analysis was conducted using one‐way ANOVA, followed by Tukey's post hoc test (a, c, d) and Student's *t* test (b). Different letters indicate statistically significant differences (*p* < 0,05). The experiments were performed with a sample size, and each experiment was replicated.

The SHEDs were treated with varying concentrations of YM extract (up to 200 μg/mL) over 1, 3 and 5 days to evaluate cell viability (Figure 3a). The results demonstrated a concentration‐dependent effect. On Day 1, cytotoxicity was observed only at 200 μg/mL (*p* < 0.0001), while 50 μg/mL showed the highest cell viability (~200% of control, *p* < 0.0001). On Day 3, cytotoxicity remained confined to 200 μg/mL (*p* < 0.0001), with 50 μg/mL maintaining strong viability (~150% of control). By Day 5, cytotoxicity was evident at 100 and 200 μg/mL, whereas 50 μg/mL continued supporting superior cell survival (~125% of control, *p* < 0.05). These findings establish 50 μg/mL as the highest non‐cytotoxic concentration, consistently promoting optimal cell viability across all time points.

To investigate the effect of YM on pro‐inflammatory cytokines, the highest non‐toxic concentration from the cell viability test, 50 μg/mL, was used in cells stimulated with LPS (Figure 3b). YM treatment significantly reduced TNF‐α expression in the YM group (0.94 ± 0.24 pg/mL) compared to the LPS group (1.57 ± 0.47 pg/mL, *p* = 0.0146). Similarly, IL‐1α levels were significantly lower in the YM group (0.77 ± 0.09 pg/mL) than in the LPS group (1.42 ± 0.26 pg/mL, *p* = 0.0002). In contrast, IL‐6 levels were comparable between the two groups, with the LPS group showing 3.57 ± 0.76 pg/mL and the YM group showing 3.62 ± 0.31 pg/mL, with no significant difference (*p* = 0.9104). These results suggest that YM effectively reduces TNF‐α and IL‐1α without adversely affecting IL‐6 expression.

The NF‐κB pathway regulates gene expression in response to pathogenic stimuli, influencing inflammation, immune responses and cell proliferation. To evaluate the impact of the YM extract on NF‐κB activity, an NF‐κB reporter assay was employed. After 6 h of LPS stimulation, NF‐κB transcriptional activity was significantly increased in the LPS group (19.80 ± 1.00) compared to the control group (13.60 ± 0.50, *p* < 0.0001). Importantly, YM at 50 μg/mL did not activate NF‐κB in the absence of LPS, indicating no pro‐inflammatory effect. However, when combined with LPS, YM significantly reduced NF‐κB activity (15.50 ± 0.56) compared to the LPS‐only group (*p* = 0.0001), as shown in Figure 3c. These findings suggest that YM can attenuate LPS‐induced NF‐κB activation without triggering the pathway under non‐inflammatory conditions. Moreover, adding YM extract at a 50 μg/mL concentration in RANKL‐treated cells demonstrated a significant inhibitory effect on osteoclastogenesis, a critical process in bone resorption associated with AP. The YM group exhibited a significant reduction in the number of TRAP‐positive osteoclasts (37.71 ± 7.60) compared to the control (55.88 ± 7.55, *p* < 0.05), as shown in Figure 3d,e.

### 3.2 Yerba Mate Restores Antioxidant Capacity and Reduces Redox State In Vivo

**FIGURE 4:**
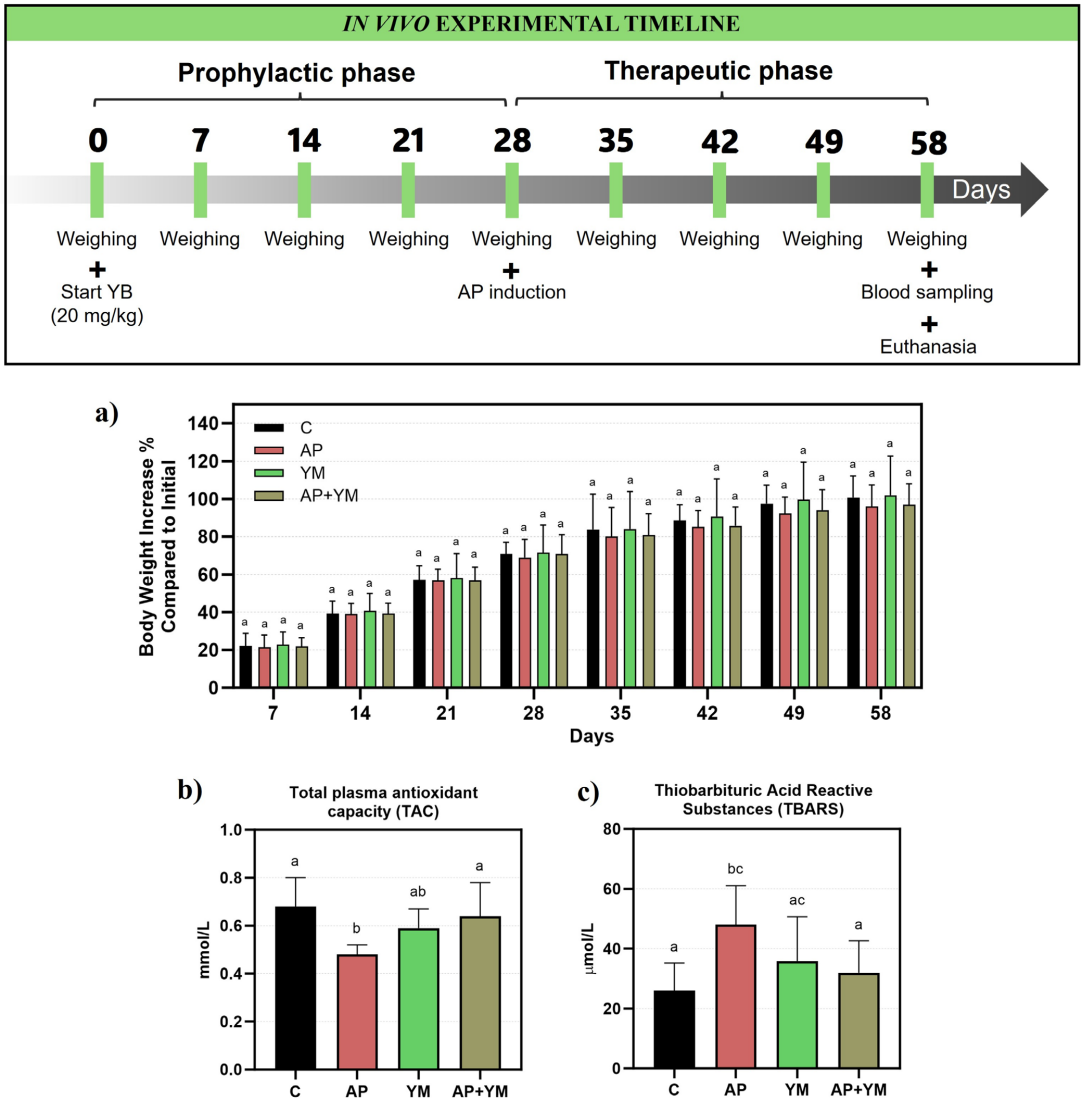
YM treatment mitigates the systemic effects of AP in vivo. Experimental timeline: Day 0 marks the start of YM treatment, day 28 corresponds to AP induction and day 58 indicates blood sampling and euthanasia. (a) Body weight changes: There were no statistically significant differences in the rate of body weight gain among the groups. (b) Total plasma antioxidant capacity (TAC): YM treatment restored the reduction in TAC caused by AP. (c) Lipid peroxidation: The AP group exhibited increased lipid peroxidation, as measured by TBARS. In contrast, YM treatment in the AP + YM group reversed this effect. Data are presented as mean ± standard deviation. Statistical analysis was performed using one‐way or two‐way ANOVA, followed by Tukey's post hoc test, with a significance level of 5%. Different letters indicate statistically significant differences. The sample size was *n* = 10 per group. C: Control; AP: Apical periodontitis; YM: Yerba Mate.

The body weight percentage gain of the animals demonstrated a consistent and gradual increase across all experimental groups throughout the study. No statistically significant differences were observed between the C, AP, YM or AP + YM groups at any time point (*p* > 0.05), indicating that neither the induction of AP nor the treatment of YM had an adverse effect on the animals' overall growth and weight gain (Figure 4a). The total plasma antioxidant capacity (TAC) analysis highlighted the systemic effects of AP on redox state. AP significantly reduced plasma antioxidant capacity (0.48 ± 0.04 mmol/L) compared to the control group (0.68 ± 0.12 mmol/L, *p* < 0.001), indicating impaired systemic antioxidant defences. However, treatment with YM in the AP + YM group effectively restored antioxidant capacity (0.64 ± 0.14 mmol/L), bringing the TAC levels close to those observed in the control group and significantly higher than the AP group alone (*p* < 0.001), suggesting a protective effect against AP‐induced redox state evoked by the YM (Figure 4b).

Furthermore, the assessment of thiobarbituric acid reactive substances (TBARS) also revealed the systemic impact of AP on oxidative stress by measuring lipid peroxidation levels. The AP group showed significantly elevated TBARS levels (48.03 ± 13.03 μmol/L) compared to the control group (25.99 ± 9.21 μmol/L, *p* < 0.001), indicating increased lipid oxidative damage. Notably, YM treatment in the AP + YM group significantly reduced TBARS levels (31.91 ± 10.65 μmol/L), bringing them closer to those observed in the control group (*p* < 0.05), mitigating the AP‐induced lipid peroxidation (Figure 4c).

### 3.3 Immunomodulatory Effects of Yerba Mate in Apical Periodontitis

**FIGURE 5:**
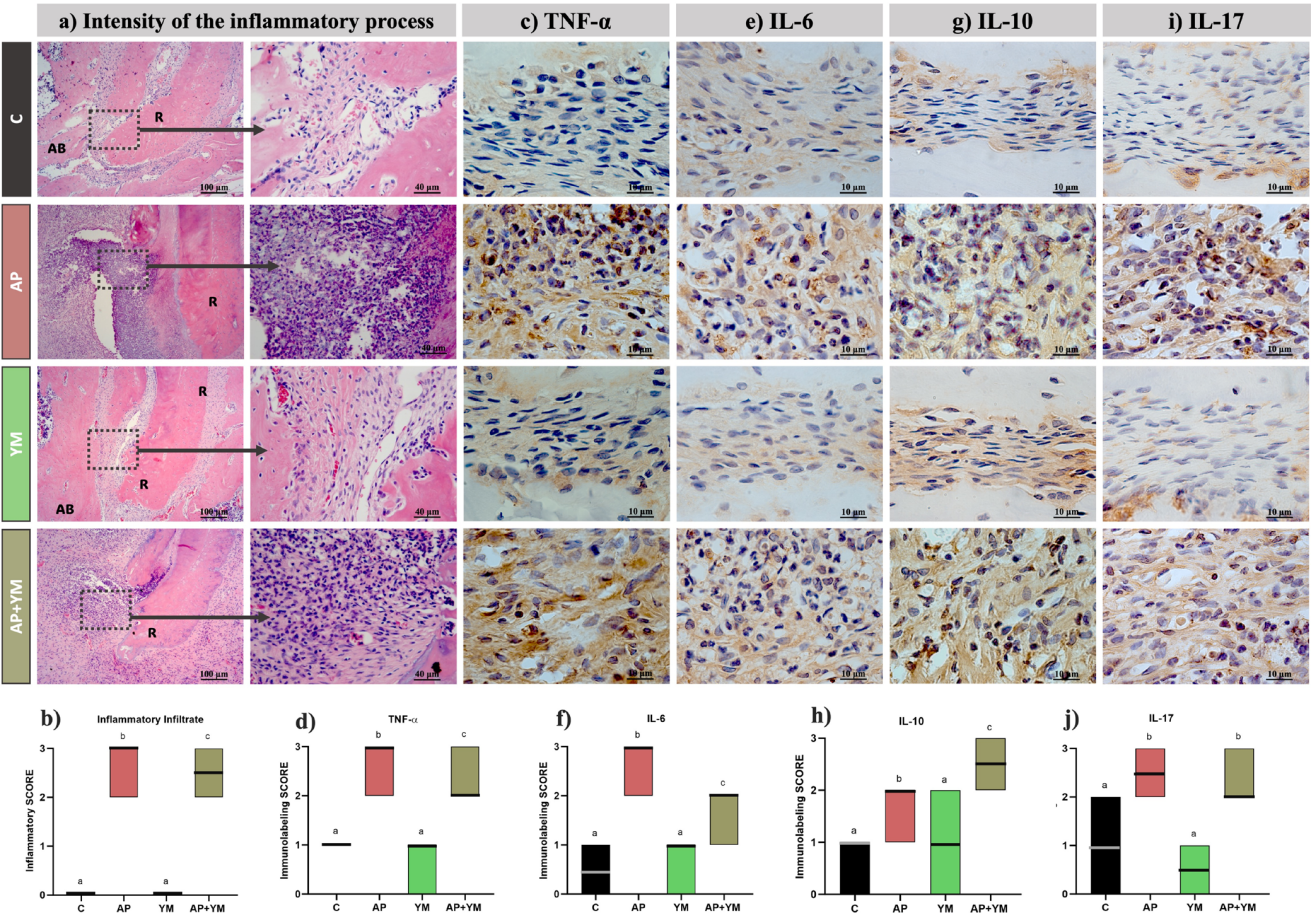
Local effects of YM on the inflammatory process of AP in vivo. Representative histological sections stained with haematoxylin and eosin (a) and immunohistochemistry for TNF‐α (c), IL‐6 (e), IL‐10 (g) and IL‐17 (i) are shown for the apex of the distal root of the lower right first molar. Inflammatory infiltrate scores (b) revealed that the AP + YM group exhibited significantly less inflammation than the AP group. Immunolabelling scores demonstrated higher TNF‐α (f) and IL‐6 (f) expression in the AP group compared to the AP + YM group. In contrast, the AP + YM group showed significantly higher IL‐10 immunostaining (h), indicating YM's anti‐inflammatory effects. No significant differences in IL‐17 levels were observed between the AP and AP + YM groups (j). In the figure, “R” denotes the root, and “AB” denotes the alveolar bone. Data are presented as mean ± standard deviation. Statistical analysis was performed using the Kruskal‐Wallis test, followed by Dunn's post hoc test at a significance level of 5%. Different letters indicate statistically significant differences. Strong black or grey lines in the graphs indicate the median. The sample size was *n* = 10 per group.

Representative histological images of inflammatory infiltrate and cytokine immunolabelling for all groups are shown in Figure 5. The intensity of the inflammatory infiltrate was analysed 30 days after AP induction to evaluate the effects of YM on the severity of AP. The C and YM groups exhibited healthy histological structures, characterised by normal pulp and periapical tissues. In contrast, subjected to induced AP, the AP and AP + YM groups exhibited histological features consistent with chronic AP lesions, including necrotic pulp, disorganised periodontal ligament, inflammatory infiltrate and extensive bone resorption. Notably, YM treatment in the AP + YM group significantly reduced the severity of the periapical inflammatory infiltrate compared to the AP group (*p* < 0.001), indicating its anti‐inflammatory potential (Figure 5a,b).

To evaluate the immunomodulatory effects of YM in AP, cytokine immunolabelling was assessed 30 days post‐induction. Immunolabelling patterns for TNF‐α (Figure 5c,d) indicated that the control and YM groups exhibited lower immunolabelling scores (median 1) than those with lesions. Notably, the AP group demonstrated significantly higher TNF‐α immunolabelling (median 3) than the AP + YM group (median 2, *p* = 0.006). Similarly, for IL‐6 immunolabelling (Figure 5e,f), the C and YM groups had lower scores compared to lesion groups, while the AP group displayed significantly higher immunolabelling (median 3) than the AP + YM group (median 2, *p* < 0.001). In contrast, IL‐10 immunolabelling (Figure 5g,h), a marker of anti‐inflammatory activity, was lower in the C and YM groups (median 1) compared to the lesion groups. However, the AP + YM group showed increased immunolabelling (median 2.5) relative to the AP group (median 2, *p* = 0.005). For IL‐17 (Figure 5i,j), the C and YM groups also exhibited lower immunostaining than the lesion groups. However, no significant difference was observed between the AP and AP + YM groups (*p* = 0.188).

### 3.4 Bone‐Protective Effects of Yerba Mate in Apical Periodontitis

**FIGURE 6:**
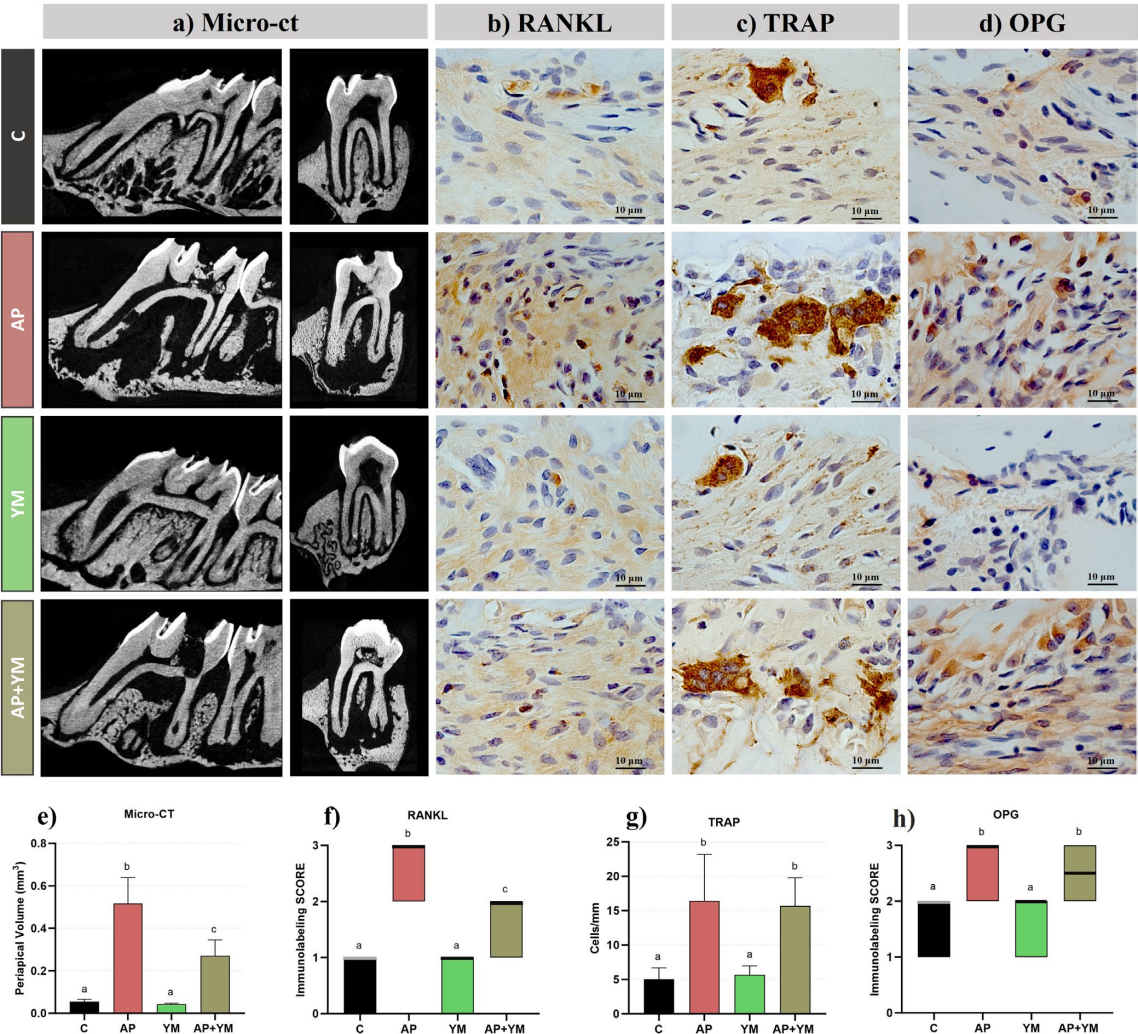
Local effects of YM on the bone resorption process of AP in vivo. Representative micro‐CT images of the distal root of the upper right first molar, including sagittal and coronal sections (a), and immunohistochemistry for RANKL (c), TRAP (e) and OPG (g) demonstrate the effects of YM. Micro‐CT analysis revealed that AP was significantly larger in the AP group compared to the AP + YM group (b), highlighting YM's protective effect against bone resorption. Immunolabelling showed higher RANKL expression in the AP group compared to the AP + YM group (d). Additionally, both AP and AP + YM groups exhibited higher TRAP and OPG immunolabelling than the control (C) and YM‐alone groups (f–g). Data are presented as mean ± standard deviation. Statistical analysis was performed using one‐way ANOVA followed by Tukey's post hoc test at a 5% significance level. Different letters indicate statistically significant differences. Strong black or grey lines in the graphs indicate the median. The sample size was *n* = 10 per group.

In the micro‐CT analysis (Figure 6a,b) of the right maxilla, the periodontal ligament areas served as the analysis sites for the C and YM groups, as these groups did not exhibit periapical lesions. The measured volumes were C (0.05 ± 0.01 mm^3^) and YM (0.04 ± 0.004 mm^3^), reflecting structural integrity at these sites. In contrast, the AP (0.51 ± 0.12 mm^3^) and AP + YM (0.27 ± 0.07 mm^3^) groups demonstrated significant alveolar bone loss in the periapical region of the roots of the maxillary first molars. Notably, the AP group exhibited more significant alveolar bone loss than the AP + YM group (*p* = 0.008).

For RANKL immunolabelling (Figure 6c,d), the C and YM groups exhibited lower immunolabelling scores (median 1) than those with lesions. Conversely, the AP group showed significantly higher RANKL immunolabelling (score 3) compared to the AP + YM group (score 2, *p* < 0.001). The number of TRAP‐positive multinucleated cells per μm along the alveolar bone surface perimeter (Figure 6e,f) was similar between the C (5.06 ± 1.62) and YM (5.68 ± 1.27) groups (*p* > 0.05). Likewise, no significant difference was observed between the AP (16.44 ± 6.77) and AP + YM (15.68 ± 4.11) groups (*p* = 0.419). For OPG immunolabelling (Figure 6g,h), the C and YM groups had lower scores (median 2) than the lesion groups. However, no statistical difference was found between the AP and AP + YM groups (*p* = 0.609).

## 4 Discussion

This study is the first to investigate the immunomodulatory and bone‐protective effects of YM using both in vitro oral‐derived stem cell models and in vivo models with systemic treatment via oral gavage. Our findings demonstrate that YM can mitigate systemic oxidative stress and the local severity of the AP. In the in vitro analysis, YM doses up to 50 μg/mL supported cell viability, while higher doses of 100 and 200 μg/mL reduced viability. This reduction can be attributed to the high concentrations of antioxidant compounds in YM, such as polyphenols and saponins, which at elevated levels may exert pro‐oxidative effects and cytotoxic potential (Puangpraphant and de Mejia 2009; Pereira et al. 2017).

In addition to demonstrating cytocompatibility, YM exhibited significant immunomodulatory potential both in vitro and in vivo. In vitro, YM's anti‐inflammatory effects were observed through a luciferase assay that assessed the activation of the NF‐κB signalling pathway. Cells treated with YM and stimulated with LPS exhibited reduced activation of this pathway compared to cells stimulated with LPS alone, indicating that YM contributes to mitigating immunological and inflammatory processes by decreasing the production of pro‐inflammatory cytokines. We specifically observed a reduction in TNF‐α levels in SHED cells stimulated with LPS, a pro‐inflammatory cytokine predominantly released by macrophages and critically involved in periodontitis‐associated bone loss. Furthermore, YM extract decreased IL‐1α expression in the same cell lineage and stimulation, reinforcing its anti‐inflammatory properties and highlighting its potential therapeutic value.

The oral gavage method was chosen for the in vivo model to simulate human consumption of YM. For this, a dose of 20 mg/kg/day, equivalent to approximately 300 mL (1.5 cups of tea) per day in humans, was used (Pereira et al. 2017). The animals tolerated this dosage well, with no changes in food intake and consistent weight gain observed. YM was administered prophylactically for 28 days before AP induction, replicating its everyday use. After AP lesions were induced, YM was given therapeutically for another 30 days to evaluate its effects on AP lesions systemically and locally (Brasilino et al. 2018). Additionally, the 30‐day period used for pulp necrosis induction and exposure to the oral environment was sufficient to allow for the development of AP, characterised by granulomatous tissue formation and inflammatory infiltrate, as supported by previous studies (Cintra et al. 2016).

Inflamed pulps exhibit elevated levels of antioxidant enzymes such as catalase and superoxide dismutase, along with increased malondialdehyde and other lipid peroxidation byproducts (Esposito et al. 2003). Previous research on chronic AP has shown that disease‐related systemic oxidative stress increases lipid peroxidation, diminishes non‐enzymatic antioxidant defences in the gastrocnemius muscle and can even lead to insulin resistance. These findings reinforce that AP is not confined to the oral cavity but can trigger metabolic and inflammatory alterations in distant organs and systems (Tsosura et al. 2021; Dos Santos et al. 2023). For example, animals with AP also showed significantly higher oxidative degradation of plasma fatty acids, as indicated by the TBARS analysis. This unregulated oxidative stress leads to the degradation of plasma membrane lipids (Hauck and Bernlohr 2016), causing structural and permeability alterations that ultimately result in cell death (Phaniendra et al. 2015). However, animals with AP lesions treated with YM reversed these effects, with TBARS levels returning to normal and aligning closely with those of the control group. These findings align with previous studies, which have shown that YM treatment reduces thiobarbituric acid‐reactive substances in the liver and blood, suggesting that YM protects unsaturated fatty acids from oxidation and mitigates oxidative damage (Martins et al. 2009).

Moreover, animals with AP exhibited a reduced total antioxidant capacity in plasma. This reduction occurs because AP increases the synthesis of reactive oxygen species (ROS) and the expression and activation of matrix metalloproteinases (MMPs) (Hernandez‐Rios et al. 2017). This redox imbalance caused by AP lesions leads to oxidative stress, disrupting bone homeostasis and disorganising periapical tissue (Hernandez‐Rios et al. 2017). However, YM treatment in animals with AP reversed these effects, restoring plasma antioxidant capacity to levels comparable to those of the control group, emphasising YM's potent antioxidant properties, primarily attributed to its phenolic compounds, particularly chlorogenic acids and flavonoids (Baeza et al. 2016). Flavonoids act as antioxidants by neutralising free radicals in both lipophilic and hydrophilic cellular compartments, and they also inhibit chain reactions triggered by free radicals (Bixby et al. 2005).

In addition to reducing lipid peroxidation and enhancing systemic antioxidant defences, YM demonstrated a local anti‐inflammatory effect by decreasing the inflammatory infiltrate caused by pulp exposure compared to the group with AP lesions alone. Animals with AP lesions treated with YM showed reduced TNF‐α immunolabelling compared to those with AP alone. YM also significantly decreased IL‐6 expression, a cytokine linked to infections and tissue damage (Tanaka et al. 2018), while increasing IL‐10 levels in AP lesions. IL‐10 is a crucial anti‐inflammatory cytokine that suppresses the release of IL‐1, IL‐6 and TNF‐α, reduces nitric oxide production and inhibits collagenase activity (Akdis et al. 2016). Studies attribute YM's anti‐inflammatory effects primarily to quercetin, a component identified as a potent inhibitor of inflammation with the ability to block COX‐2 (Puangpraphant and de Mejia 2009). Additionally, YM's combination of quercetin with saponins results in a synergistic effect, further inhibiting nitric oxide and prostaglandin 2 production (Burris et al. 2012). The reduction in the severity of periapical lesions may also be related to the antimicrobial action of YM, because it contains one or more antimicrobial compounds, such as secondary metabolites derived from chlorogenic acid (Rempe et al. 2015). Research has shown that YM is a potent bactericide and inhibitor of the growth of bacterial pathogens such as *Staphylococcus aureus* (Kubo et al. 1993; Burris et al. 2012; Prado Martin et al. 2013), *Enterobacter cloacae, Streptococcus mutans, Listeria monocytogenes, Salmonella enteritidis* (Burris et al. 2012; Prado Martin et al. 2013) and *Enterococcus faecalis* (Girolometto et al. 2009).

The RANK/RANKL/OPG system is a central signalling pathway regulating osteoblast and osteoclast activity (Silva and Branco 2011). OPG, with its osteoprotective properties, binds to RANKL, preventing its interaction with RANK and inhibiting osteoclast formation (Silva and Branco 2011). In the context of YM's bone‐protective effects in AP, animals with AP lesions without YM treatment exhibited significantly higher RANKL immunolabelling than those receiving YM. Although there were no statistical differences in OPG immunolabelling or TRAP levels, micro‐CT analysis revealed less alveolar bone loss in YM‐treated groups than in those not treated. This reduction in bone resorption may be attributed to the higher RANKL activity observed in animals without YM treatment, which promotes osteoclastic activity. Additionally, the results suggest a complementary pathway influencing osteoclast formation in vivo since our in vitro osteoclastogenesis findings demonstrated YM's potential to modulate osteoclast activity. This can be attributed to decreased TNF‐α levels, which play a critical role in osteoclast recruitment and bone loss, alongside increased IL‐10 expression, a key cytokine for bone homeostasis that prevents bone loss by upregulating OPG expression and downregulating RANKL expression, thereby inhibiting osteoclast activation and differentiation (Kitaura et al. 2014).

The beneficial effects on bone metabolism may be attributed to the synergistic action of yerba mate's components, particularly its antioxidant properties, which have already been proven to positively influence bone metabolism (Zhang et al. 2011; Shen et al. 2013). In a recent study, YM components, including rutin, chlorogenic acid and caffeine, demonstrated positive effects on bone cells, particularly pre‐osteoclast cells (MC3TC‐E1), increasing their viability and cell differentiation (Villarreal et al. 2024). Another compound, chlorogenic acid, has been shown to have a promoting effect on osteoblast differentiation, leading to greater osteogenic activity (Zhou et al. 2016; Clough et al. 2017). It has also prevented RANKL‐induced osteoclastogenesis (Kwak et al. 2013) and promoted the osteogenic differentiation of human dental pulp stem cells through the Wnt signalling pathway (Hu et al. 2021). Theobromine has also been shown to increase alkaline phosphatase activity and osteoprotegerin levels, as well as promote the mineralisation of primary human bone marrow‐derived mesenchymal stem cells (Clough et al. 2017). Additionally, quercetin contributed to osteoblast differentiation by inhibiting nuclear factor‐kappa B (NF‐κB) activation and TNF‐α‐induced degradation of β‐catenin in rat BM‐MSCs. Other studies have also shown the beneficial effects of YM on bone metabolism in animal models, where YM treatment reduced bone resorption in rats during the perimenopausal period, attenuating the deterioration of bone microarchitecture, observing an increase in trabecular area, the number of osteocytes and bone mineral density, along with a reduction in malondialdehyde levels in femoral tissue, as well as a decrease in plasma levels of tartrate‐resistant acid phosphatase (TRAP), a classic marker of osteoclastic activity (Pereira et al. 2017). In addition, in another study, YM improved bone quality in ovariectomised rats and promoted osteoblastic differentiation (Zhou et al. 2016; Clough et al. 2017; Balera Brito et al. 2019).

Our study has methodological limitations. First, only non‐enzymatic antioxidant defence, CAT, was evaluated, so analyses of enzymatic antioxidant defences, such as superoxide dismutase (SOD) and determination of reduced glutathione (GSH) (Dos Santos et al. 2023) are also important to provide a more in‐depth assessment of the antioxidant effect observed with YM treatment. Second, in addition to CAT and TBARS, AP is also associated with changes in other biomarkers, such as increased synthesis of reactive oxygen species (ROS), increased expression and activation of matrix metalloproteinases (MMPs) (Hernandez‐Rios et al. 2017) and increased antioxidant enzyme catalase (CAT) (Esposito et al. 2003). Furthermore, analyses of haematological parameters are relevant, as AP has been shown to increase leukocyte and lymphocyte counts, as well as serum levels of cytokines such as TNF‐α (Samuel et al. 2018).

Overall, this study provides encouraging evidence for the systemic consumption of YM; however, its broader systemic effects, particularly its antioxidant activity, remain unclear. Translating in vitro and in vivo findings to clinical practice requires caution. Animal models remain essential for elucidating the pathogenesis of AP and for evaluating adjunctive endodontic therapies. Nevertheless, no clinical trials have assessed the effect of YM treatment on AP in humans. Additional research should therefore examine its systemic actions in greater depth and explore optimal dosing and alternative delivery strategies.

## 5 Conclusion

In vitro, YM extract demonstrated cellular compatibility and immunomodulatory effects by reducing pro‐inflammatory cytokines (TNF‐α and IL‐1α), attenuating NF‐κB pathway activation and inhibiting osteoclastogenesis. In vivo, YM treatment reversed AP‐induced changes in systemic redox state and lipid peroxidation. Furthermore, in AP lesions, YM treatment decreased the local inflammatory response and reduced bone resorptive activity.

## Author Contributions

**Carolina Sayuri Wajima:** conceptualisation, data curation, methodology, investigation, writing – original draft, funding acquisition. **Carolina de Barros Morais Cardoso:** conceptualisation, methodology, investigation, writing – original draft. **Caroline Anselmi:** conceptualisation, data curation, methodology, investigation, writing – original draft. **Renan Dal‐Fabbro:** conceptualisation, data curation, formal analysis, methodology, investigation, writing – review and editing. **Murilo Catelani Ferraz:** formal analysis, methodology, writing – original draft. **Cristiane Cantiga da Silva:** methodology, investigation, writing – original draft. **Paulo César Ciarlini:** methodology, investigation, writing – original draft. **Edilson Ervolino:** formal analysis, methodology, writing – review and editing. **Marco Cícero Bottino:** conceptualisation, formal analysis, methodology, resources, writing – review and editing, supervision, funding acquisition, project administration. **Luciano Tavares Angelo Cintra:** conceptualisation, formal analysis, methodology, resources, writing – review and editing, supervision, funding acquisition, project administration.

## Ethics Statement

This study was approved by the Ethics Committee on the Use of Animals (CEUA) at São Paulo State University (UNESP), School of Dentistry, Araçatuba, Brazil (#0197–2022, approved April 25, 2022).

## Conflicts of Interest

The authors declare no conflicts of interest.

## Data Availability

The data that support the findings of this study are available from the corresponding author upon reasonable request.
